# Spliceosomic dysregulation in pancreatic cancer uncovers splicing factors PRPF8 and RBMX as novel candidate actionable targets

**DOI:** 10.1002/1878-0261.13658

**Published:** 2024-05-24

**Authors:** Emilia Alors‐Pérez, Ricardo Blázquez‐Encinas, María Trinidad Moreno‐Montilla, Víctor García‐Vioque, Juan Manuel Jiménez‐Vacas, Andrea Mafficini, Iranzu González‐Borja, Claudio Luchini, Juan M. Sánchez‐Hidalgo, Marina E. Sánchez‐Frías, Sergio Pedraza‐Arevalo, Antonio Romero‐Ruiz, Rita T. Lawlor, Antonio Viúdez, Manuel D. Gahete, Aldo Scarpa, Álvaro Arjona‐Sánchez, Raúl M. Luque, Alejandro Ibáñez‐Costa, Justo P. Castaño

**Affiliations:** ^1^ Maimonides Biomedical Research Institute of Córdoba (IMIBIC) Spain; ^2^ Department of Cell Biology, Physiology, and Immunology University of Córdoba Spain; ^3^ Reina Sofia University Hospital Córdoba Spain; ^4^ ARC‐Net Research Centre and Section of Pathology of Department of Diagnostics and Public Health University and Hospital Trust of Verona Italy; ^5^ OncobionaTras Lab, Navarrabiomed, Hospital Universitario de Navarra, Instituto de Investigación Sanitaria de Navarra‐IDISNA Universidad Pública de Navarra Pamplona Spain; ^6^ CIBER Fisiopatología de la Obesidad y Nutrición (CIBERobn) Córdoba Spain; ^7^ Surgery Service Reina Sofia University Hospital Córdoba Spain; ^8^ Pathology Service Reina Sofia University Hospital Córdoba Spain; ^9^ ICON plc Pamplona Spain

**Keywords:** pancreatic cancer, PRPF8, RBMX, splicing, splicing factor

## Abstract

Pancreatic ductal adenocarcinoma (PDAC) is a highly lethal cancer, characterized by late diagnosis and poor treatment response. Surgery is the only curative approach, only available to early‐diagnosed patients. Current therapies have limited effects, cause severe toxicities, and minimally improve overall survival. Understanding of splicing machinery alterations in PDAC remains incomplete. Here, we comprehensively examined 59 splicing machinery components, uncovering dysregulation in pre‐mRNA processing factor 8 (*PRPF8*) and RNA‐binding motif protein X‐linked (*RBMX*). Their downregulated expression was linked to poor prognosis and malignancy features, including tumor stage, invasion and metastasis, and associated with poorer survival and the mutation of key PDAC genes. Experimental modulation of these splicing factors in pancreatic cancer cell lines reverted their expression to non‐tumor levels and resulted in decreased key tumor‐related features. These results provide evidence that the splicing machinery is altered in PDAC, wherein PRPF8 and RBMX emerge as candidate actionable therapeutic targets.

## Graphical abstract

The machinery that carries out maturation of RNA, the spliceosome, is severely altered in pancreatic ductal adenocarcinoma. Decreased expression of two splicing factors, PRPF8 and RBMX, is linked to aggressiveness features and poor survival, while their rescue improves cell behavior. Splicing dysregulation provides a promising vulnerability of pancreatic cancer that can be harnessed to identify novel diagnostic and treatment tools.

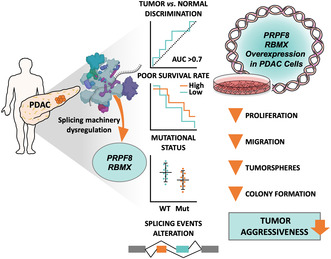

**Abbreviations**
EGF: epidermal growth factor
FBS: fetal bovine serum
FFPE: formalin‐fixed paraffin‐embedded
HS: horse serum
IHC: immunohistochemistry
KSFM: keratinocyte serum free medium
PBS: phosphate buffered saline
Pd: pladienolide B
PDAC: pancreatic ductal adenocarcinoma
PVP: polyvinylpyrrolidone
qPCR: quantitative polymerase chain reaction
SF: splicing factor
TCGA: The Cancer Genome Atlas

## 1 Introduction

Pancreatic ductal adenocarcinoma (PDAC) accounts for 90% of pancreas neoplasms and is one of the most lethal cancers worldwide, with a dismal 10% survival rate 5 years after diagnosis [1]. Despite the profound knowledge acquired in recent years on the molecular basis of PDAC [2, 3], its translation to the patient is still very limited. Accordingly, opening novel areas of research is required to tackle this disease. A growing number of studies [4, 5], including several from our group [6, 7, 8, 9, 10, 11], show that many different cancers share as a common hallmark the alteration of the splicing machinery, which leads to abnormal patterns of alternative splicing and to the rise of aberrant variants with oncogenic potential. Interestingly, PDAC was one of the first cancers where alternative splicing was explored. Pioneering studies unveiled mutations and alterations in the expression of several components of the splicing machinery and led to identify dysregulated profiles of splice variants [12, 13, 14]. Thus, functional and bioinformatic studies in PDAC have provided evidence for the relevance of specific alterations in splicing machinery components, both spliceosome core elements and splicing factors. Notable examples include *SRPK1* and *SRSF1*, whose relation to tumor progression and gemcitabine resistance was suggested by a study in PDAC cell lines [15, 16]; and *RBM5*, which was reported to be correlated with *KRAS* expression and several clinical parameters in PDAC, suggesting its involvement in tumor invasion and progression [17]. Likewise, *ESRP1* expression was related to longer overall survival and lower grading tumors [18]. Most recently, our group discovered that *SF3B1* is overexpressed in PDAC, where it imparts malignancy features but also unveiling a dual therapeutic vulnerability, as its function can be targeted in pancreatic cancer cells and cancer stem cells with an anti‐splicing drug, Pladienolide B [10]. Moreover, the splicing factor *RBFOX2* was recently identified as a metastatic suppressor in PDAC [19].

Taking this evidence together, we posited that the alterations found in individual factors may indicate that the splicing machinery is uniquely and profoundly dysregulated in PDAC, and that its systematic study could help to identify further potential biomarkers and operable tools. Accordingly, in the present study, we deployed a strategy to explore the expression of the components of the spliceosome core and a selected set of splicing factors in three PDAC cohorts, assess their relation to clinical/molecular parameters, and study key functional and pathological features.

## 2 Materials and methods

### 2.1 Patients and samples

This study was performed using a cohort of 76 patients diagnosed with PDAC which were collected from March 2017 to January 2021 at the Reina Sofia University Hospital (Córdoba, Spain). The tumors were resected, formalin‐fixed and paraffin‐embedded (FFPE), and all samples were histologically examined by expert pathologists to detect and obtain portions of tumor tissue and non‐tumor adjacent tissue from each case. Clinical parameters were collected to carry out association analyses [10]. This study was approved by the Ethics Committee of the Reina Sofia University Hospital (Cordoba, Spain; protocol CANPANC‐HYC‐01, v2, 14/06/2016), and was conducted in accordance with the Declaration of Helsinki and with national and international guidelines. A written informed consent was signed by each patient. A second cohort including 195 PDAC and 41 non‐neoplastic pancreas samples from Jandaghi *et al*. [20] was used to confirm differential expression of splicing factors in neoplastic vs. non‐neoplastic pancreas. Data were obtained from dataset E‐MTAB‐1791 of the public database “ArrayExpress” (www.ebi.ac.uk/arrayexpress/). A third cohort of 177 Pancreatic Adenocarcinoma patients from the PanCancer study was used as validation cohort (The Cancer Genome Atlas dataset; https://portal.gdc.cancer.gov/). Clinical and RSEM normalized mRNA expression data from these patients were downloaded from cBioPortal [21, 22] for further analyses.

### 2.2 Gene expression and splicing variants analysis

RNA‐seq data produced from a fourth cohort of 94 PDAC samples (“Verona cohort”, cohort 4, see Table 1) were analyzed to explore splicing profiles according to either PRPF8 or RBMX expression. The dataset and the analysis workflow was previously described in detail [10]. The difference in average PSI from each group with adjusted, and *P* < 0.01 were considered significant.

**Table 1:** Overview of the data cohorts studied and analyses performed within those cohorts.

	Cohort 1	Cohort 2	Cohort 3	Cohort 4	Cell lines
Number of samples	146	236	177	94	BxPC‐3
Non‐tumor tissue	70	41	0	0	
Tumor tissue	76	195	177	94	
Data available	Clinical parameters	No clinical parameters	Clinical parameters	No clinical parameters	
	No mutational data	No mutational data	Mutational data	No mutational data	Capan‐2
Data origin	In‐house samples (Córdoba)	Published dataset (Jandaghi *et al*.)	Published dataset (TCGA)	In‐house samples (Verona)	
Analyses performed	Spliceosome expression analysis (PDAC vs. normal)	Validation of *PRPF8* and *RBMX* expression (PDAC vs. normal)	*PRPF8* and *RBMX* expression vs. clinical parameters	Transcriptome analysis of splicing variants according to *PRPF8* and *RBMX* expression	Restored *PRPF8* and *RBMX* expression
	Tumor vs. normal discrimination (ROC)		*PRPF8* and *RBMX* expression vs. mutational data		Proliferation and invasion assays
	Selection of PRPF8 and RBMX				

### 2.3 RNA extraction and reverse transcription

Total RNA was extracted from FFPE samples using Maxwell MDx 16 Instrument (Promega, Madrid, Spain) with the Maxwell 16 LEV RNA FFPE Kit (Promega), following manufacturer instructions. From cell lines, RNA was isolated using TRIzol Reagent (Invitrogen, Barcelona, Spain) and treated with DNase (Promega). In both cases, RNA was quantified using NanoDrop2000 Spectrophotometer (Thermo Fisher Scientific, Madrid, Spain) and 1 μg of RNA was retrotranscribed to complementary DNA (cDNA) using random hexamer primers and the RevertAid RT Reverse Transcription Kit (Thermo Fisher Scientific).

### 2.4 qPCR dynamic array based on microfluidic technology

A quantitative PCR dynamic array based on microfluidic technology was used to simultaneously measure the expression of 96 genes in 96 PDAC tumor samples and adjacent non‐tumor samples, as previously reported [10]. Biomark System and FluidigmVR Real‐Time PCR Analysis Software v.3.0.2 and Data Collection Software v.3.1.2 (Fluidigm, San Francisco, CA, USA) were used to obtain RNA expression levels. Primers for specific human genes were designed with primer3 and primer blast software [23, 24]. RNA expression levels were normalized using the β‐actin housekeeping gene (*ACTB*).

### 2.5 Quantitative real‐time PCR (qPCR)

Quantitative PCR was performed to assess RNA expression levels in cell lines using the Brilliant III SYBR Green QPCR Master Mix (Stratagene, La Jolla, CA, USA). Each reaction was assembled using 10 μL of SYBR Green, 8.4 μL of Water, 0.3 μL of each primer and 1 μL of cDNA (50 ng·μL^−1^). The reactions were performed using the Stratagene Mx3000p system and the previously reported thermal profile [10].

### 2.6 Western blot

Western blot was performed to quantify protein expression levels in cell lines using a protocol previously reported by our group [10]. Membranes were incubated with antibodies for PRPF8 (Abcam, Cambridge, UK, #ab79237), RBMX (Invitrogen, #PA5‐99433) and beta tubulin (TUBB, Cell Signaling, Danvers, MA, USA, #2128). Then, membranes were incubated with secondary anti‐rabbit antibody (Cell Signaling, #7074S) and protein expression was quantified using Clarity Max Western ECL Substrate kit (Bio‐Rad, Hercules, CA, USA, #1705062) and ImageQuant Las 4000 system (GE Healthcare Europe GmbH, Madrid, Spain). Images were analyzed using imagej‐1.51s software (Bethesda, MD, USA). PRPF8 and RBMX expression were normalized using TUBB expression.

### 2.7 Cell culture

Cell lines used in this study included Capan‐2 (RRID:CVCL_0026) and BxPC‐3 (RRID:CVCL_0186) (ATCC, Barcelona, Spain). Capan‐2 cell line was purchased in 2021, and BxPC‐3 in 2022, and were validated by analysis of short tandem repeats (GenePrint 10 System; Promega, Barcelona, Spain). All cell lines were grown in a 37 °C atmosphere with 5% CO_2_ and constant humidity, and cultured according to the supplier's recommendations of passages < 10, and checked for mycoplasma contamination as previously reported [25]. Capan‐2 were cultured in McCoy's 5A Medium (Gibco, Madrid, Spain) supplemented with 10% fetal bovine serum (FBS, Sigma‐Aldrich, Madrid, Spain), 2 mm L‐glutamine (Sigma‐Aldrich) and 0.2% antibiotic‐antimycotic (Gentamicin/Amphotericin B; Life Technologies, Barcelona, Spain). BxPC‐3 were cultured in RPMI 1640 medium (Lonza, Basel, Switzerland) supplemented with 10% FBS, 2 mm L‐glutamine and 0.2% antibiotic‐antimycotic (Gentamicin/Amphotericin B; Life Technologies).

### 2.8 Transfection with specific plasmids

Expression plasmids were used to overexpress *PRPF8* (Origene, Rockville, MD, USA, #SC116070) and *RBMX* (Origene, #RC200777) in cell lines. Specifically, 150 000 cells were seeded in 6‐well plates and transfected using Lipofectamine 2000 (Invitrogen, Madrid, Spain) according to the manufacturer's instructions. Negative controls included empty pCMV6‐XL4 plasmid for *PRPF8* experiments, and empty pCMV6‐Entry plasmid for *RBMX* experiments. Cells were harvested after 48 h of transfection to seed them for transfection validation (qPCR) and carrying out functional assays.

### 2.9 Proliferation rate assay

Resazurin Assay (Canvax Biotech, Valladolid, Spain) was used to assess the effect of *PRPF8* and *RBMX* expression on cell proliferation. Specifically, 3500 transfected cells were seeded in a 96‐well plate and serum‐starved for 12 h. Cells were then provided with 10% resazurin medium and fluorescence at 590 nm was measured after 3 h with FlexStation III system (Molecular Devices, Sunnyvale, CA, USA). This was repeated at 24, 48 and 72 h.

### 2.10 Wound‐healing assay

50 000 transfected cells were seeded in a 96‐well Essen ImageLock plate (Essen BioScience, Ann Arbor, MI, USA) and grown to confluence. Scratches were then made in the plate using 96‐pin WoundMaker (Essen BioScience). An inverted microscope with a digital camera was used to take wound photos at 40× magnification at the moment of scratching and after 24 h.

### 2.11 Tumorsphere and colony formation

To assess tumorspheres formation, 1000 cells were seeded in a Corning Costar ultra‐low attachment plate (Sigma‐Aldrich) in DMEM F‐12 medium (Gibco) supplemented with EGF (20 ng·mL^−1^) and FGF (20 ng·mL^−1^) for 10 days. After this period, photographs were taken to visualize and measure the area of the resulting tumorspheres.

Colony formation assays were performed seeding 2000 transfected cells in a 6‐well plate and media were changed every 3 days for 10 days. Then, cells were fixed in the plate and stained with 6% glutaraldehyde and 0.05% crystal violet solution. Colonies (particles per well) were measured by ChemiDoc‐XRS+ System (Bio‐Rad).

### 2.12 Data analysis

Shapiro–Wilk test was used to test for groups normality, then unpaired t‐test was performed when data followed normal distribution and unpaired Mann–Whitney *U* test when not. When comparing three or more groups, one‐way ANOVA was applied for data following normal distribution and Kruskal‐Wallis test was applied when not. These tests were followed by Tukey's or Dunn's tests, respectively. Correlation tests were done using Pearson or Spearman distributions, based on data normality. Significance levels were considered when *P* < 0.05 (*), *P* < 0.01 (**), *P* < 0.001 (***), *P* < 0. Data are expressed by mean ± standard error of the mean (SEM), as fold change (log_2_) or relative levels compared with the corresponding controls (set at 100%). Statistical analyses were performed using prism 8 (GraphPad, La Jolla, CA, USA). sPLSDA and heatmaps were done using metaboanalyst v.4.0 (McGill University, Quebec, Canada). Survival analyses were done using R v4.0.2 and package *survminer* (https://cran.r‐project.org/package=survminer). Migration, tumorsphere, and colony assays' photos were analyzed using imagej software v.1.8.0_172.

## 3 Results

### 3.1 The pattern of expression of the splicing machinery is severely altered in PDAC

The comprehensive and robust profiling of the splicing machinery status in PDAC pursued in this study led to an extensive volume of data (4 cohorts with more than 500 tumor samples) and a remarkable diversity of data, which are summarized in Table 1 to facilitate the follow‐up of our workflow (Table 1).

**Fig. 1:**
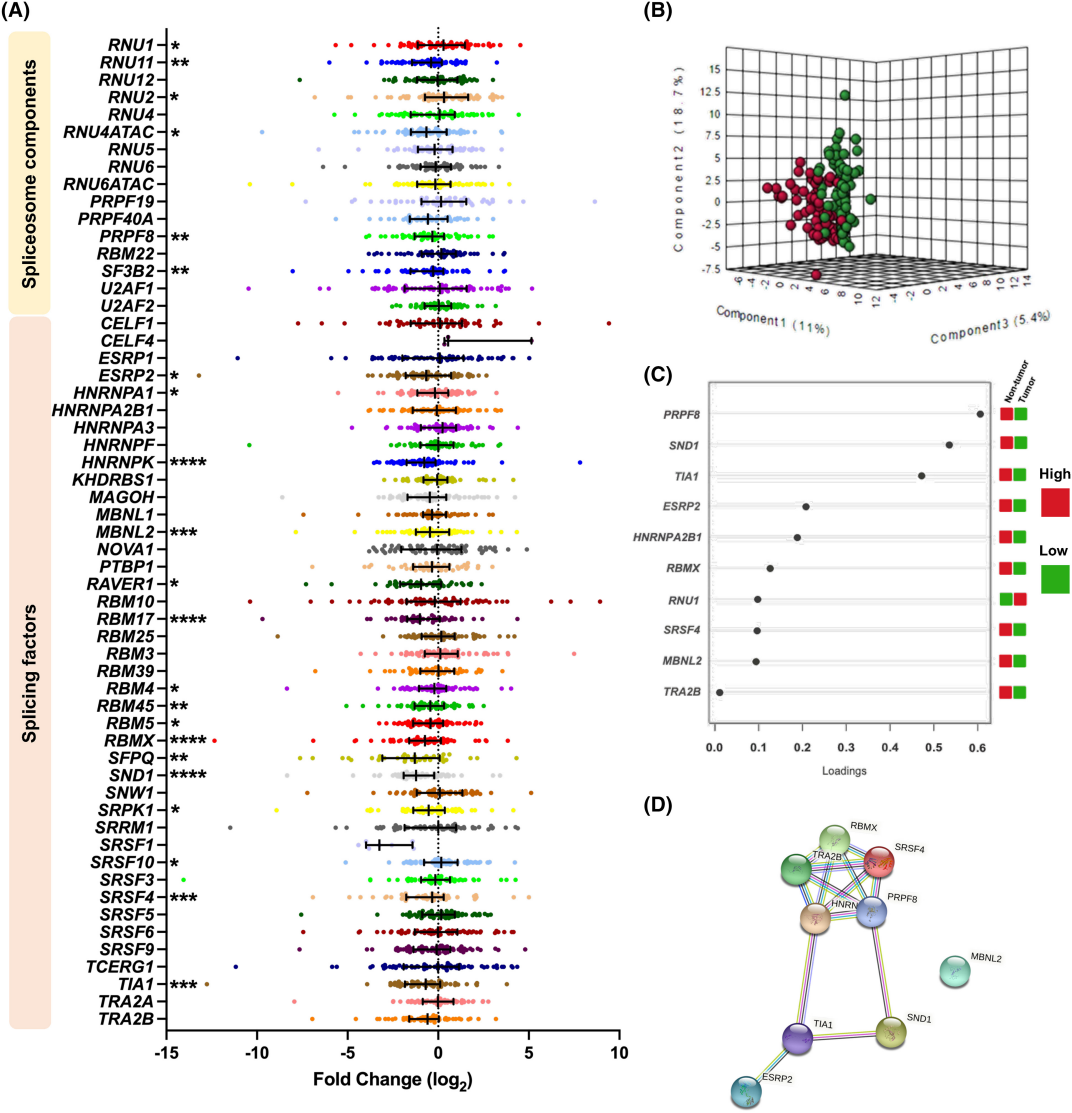
Splicing dysregulation in Pancreatic Ductal Adenocarcinoma. (A) Fold Change of mRNA levels expressions of Spliceosome Components and Splicing Factors of PDAC FFPE samples compared with non‐tumor adjacent tissue. Data are represented by Fold Change mRNA levels normalized by ACTB expression levels ± SEM. Asterisks indicate significantly differences between groups by Mann–Whitney *U* test (*P < 0.05; **P < 0.01; ***P < 0.001; ****P < 0.0001). (B) Principal Components Analysis (PCA) of the splicing machinery components analyzed in PDAC FFPE samples cohort. (C) sPLSDA analysis showing the best classifying factors between tumor and non‐tumor adjacent tissue in our cohort. Higher expression is shown in red and lower expression in green. (D) STRING analysis of relationships among altered components based on the top genes showing the most differences between the expression in tumor and non‐tumor adjacent tissues.

Results from microfluidic qPCR dynamic array revealed a clear dysregulation of splicing machinery expression in tumor vs. non‐tumor adjacent tissues in a set of 76 FFPE PDAC samples. In fact, a relevant proportion of the 16 spliceosome components and 41 splicing factors measured (38% and 39%, respectively) were differentially expressed in tumor vs. non‐tumor tissue, with a clear predominance of downregulation (Fig. 1A). Further analysis of these data was performed by applying a dedicated statistical method [26] to select among them the best predictive or discriminative elements to help classifying the tumor vs. non‐tumor tissues. As illustrated by the data distribution in the Principal Components Analysis (PCA) plot (Fig. 1B), two separate groups emerged from gene expression levels, suggesting that both sample groups could be discriminated based on the expression pattern of the splicing machinery components. Furthermore, some discernment between the two tissues becomes possible with regard to variations in gene expression for a reduced set of factors, evidencing an impairment of the physiological status of the splicing machinery in PDAC, as shown in Fig. 1C. The statistical analysis of these results was refined using Sparse Partial Least Squares Discriminant Analysis (sPLSDA) and plotting the generated loadings, which portrayed the genes with the highest ability to discriminate between tumor vs. non‐tumor adjacent tissues. As shown, when the variables were ranked by the absolute values of their loadings, the top 10 genes showing the most consistent and prominent differences between the expression in tumor and non‐tumor adjacent tissues included: *PRPF8*, *SND1*, *TIA1*, *ESRP2*, *HNRNP2AB1*, *RBMX*, *RNU1*, *SRSF4*, *MBNL2*, and *TRA2B* (Fig. 1C). Interestingly, a simple STRING analysis exploring known protein–protein interactions predicted a potential network connecting nearly all the selected genes, with a particularly tight putative cross‐regulation between *PRPF8*, *RBMX*, *HNRNP2AB1*, *SRSF4*, and *TRA2B* (Fig. 1D). Additionally, the expression pattern of a reduced set of 7 of these factors (those with the highest loading in the sPLSDA model; > 0.1) emerged as an important partial discriminating element between the subset of tumor samples and adjacent non‐tumor tissue samples (Fisher's Exact Test, *P* = 1.105e‐06) (Fig. S1A).

To gain a better understanding of the top 10 dysregulated splicing machinery components in PDAC, we inspected them in further detail. As illustrated in Fig. S1B, in this discovery cohort, tumor tissue exhibited higher levels of expression than the corresponding non‐tumor adjacent tissues in only one spliceosome component *RNU1*, whereas lower RNA levels were observed for *RBMX*, *PRPF8*, *SND1*, *TIA1*, *ESRP2*, *HNRNPA2B1*, *TRA2B*, *SRSF4*, and *MBNL2*. Furthermore, an analysis based on ROC curves indicated that all the splicing machinery components selected had an Area Under the Curve (AUC) close to or higher than 0.6, supporting their capacity to discriminate between tumor vs. non‐tumor adjacent tissues. In particular, *SND1*, *RBMX*, and *TRA2B* showed the highest discriminant ability, with AUCs above 0.7 (Fig. S1C). While single genes showed a moderate power of discrimination, an integrated ROC curve combining expression levels of the most significantly altered splicing machinery components without any weighting (*PRPF8*, *SND1*, *TIA1*, *ESRP2*, *HNRNPA2B1*, *RBMX*, *RNU1*, *SRSF4*, *MBNL2*, and *TRA2B*) yielded an AUC of 0.823 (95% CI: 0.725–0.954, Fig. S1D).

### 3.2 Splicing machinery dysregulation is associated with key clinical parameters and with distinct profiles of splicing events

Given the above results, *PRPF8* and *RBMX*, both belonging to the same tightly interactive module outlined before, were selected to be explored in further detail as they displayed markedly different expression between tumor and non‐tumor tissue (loading plots and ROC curves) and their possible role in PDAC has not been reported to date.

**Fig. 2:**
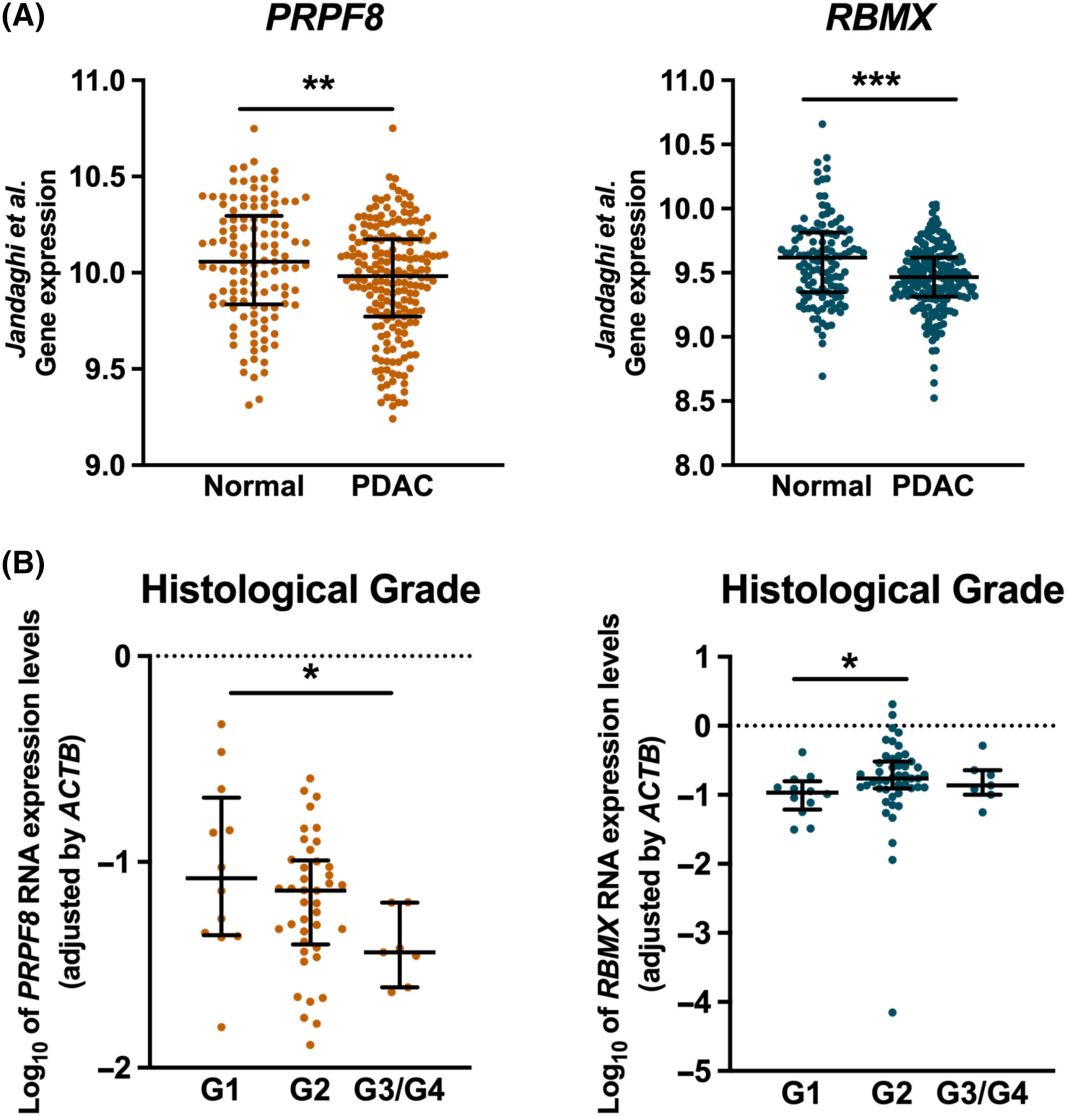
*PRPF8* and *RBMX* expression in external cohort and association with clinical parameters. (A) *PRPF8* (orange) and *RBMX* (blue) relative mRNA levels in an external validation Pancreatic Ductal Adenocarcinoma (PDAC) cohort (“Jandaghi, 2016”) [20]. Asterisks indicate significant differences between groups by Mann–Whitney *U* test (**P < 0.01; ***P < 0.001). (B) Distribution of *PRPF8* and *RBMX* Log_10_ expression levels normalized by *ACTB* expression levels in the different Histological grades of PDAC in the in‐house cohort. Median and interquartile range are represented. Asterisks indicate significant differences between groups by Dunn's test (*P < 0.05).

To validate the results obtained in the test cohort, we first carried out an *in silico* analysis of a second PDAC cohort including 195 tumors and 41 non‐tumor tissue samples obtained from the public database “ArrayExpress” (E‐MTAB‐1791). In this case, the reference tissue was obtained from healthy pancreas. Interestingly, results showed a neat parallelism with those in our cohort for both *PRPF8* and *RBMX*, which showed lower levels in tumor samples vs. normal pancreatic tissue (Fig. 2A). Interestingly, in our in‐house cohort, these two splicing factors were the only ones that displayed an association with clinical parameters, which was not appreciable for the rest of genes explored (Fig. S2). Specifically, the expression levels of both genes were associated to histological grade, although in a different manner. Indeed, *PRPF8* expression levels were inversely correlated to histological grade, being progressively lower in G1, G2, and G3/G4 PDAC samples. Conversely, *RBMX* levels were higher in G2 than G1 samples, with no apparent differences in G3/G4. These results suggest that lower *PRPF8* RNA levels, but not *RBMX* expression, are associated with more undifferentiated tumors (Fig. 2B).

**Fig. 3:**
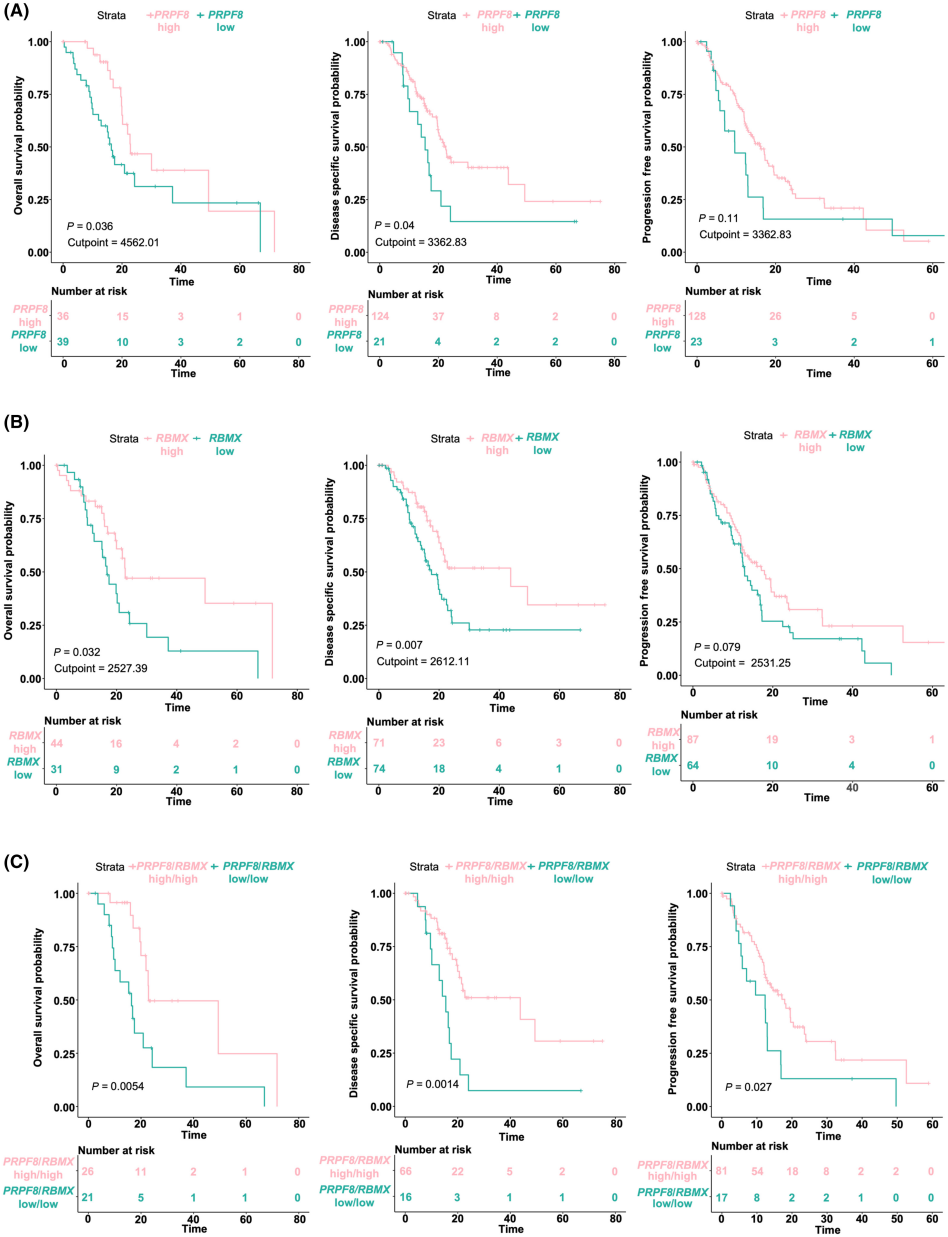
Survival analysis expression levels in PDAC. (A–C) Kaplan–Meier Survival analysis, Overall Survival and Relapse Free Survival associated with *PRPF8* (A) and *RBMX* (B), mRNA expression levels and their combination (C), respectively, in PanCancer cohort [2]. The respective curves at high (pink) and low (blue) levels of each factor are shown, as well as the *P*‐value calculated by log‐rank test, the cut‐off point to separate the expression groups and the number at risk in each group.

Analyses of patient survival parameters in relation with the expression of the two splicing elements was performed on an RNA‐seq dataset previously generated from 94 PDAC cases, which has been previously described in detail [10]. Of note, high *PRPF8* and *RBMX* expression levels were both independently associated to better patient outcome, whereas patients with low levels showed a poorer outcome, as measured by overall and disease‐specific survival (Fig. 3A,B). Accordingly, those patients exhibiting high levels of both factors displayed more favorable outcome in terms of overall, disease‐specific and progression‐free survival (Fig. 3C).

**Fig. 4:**
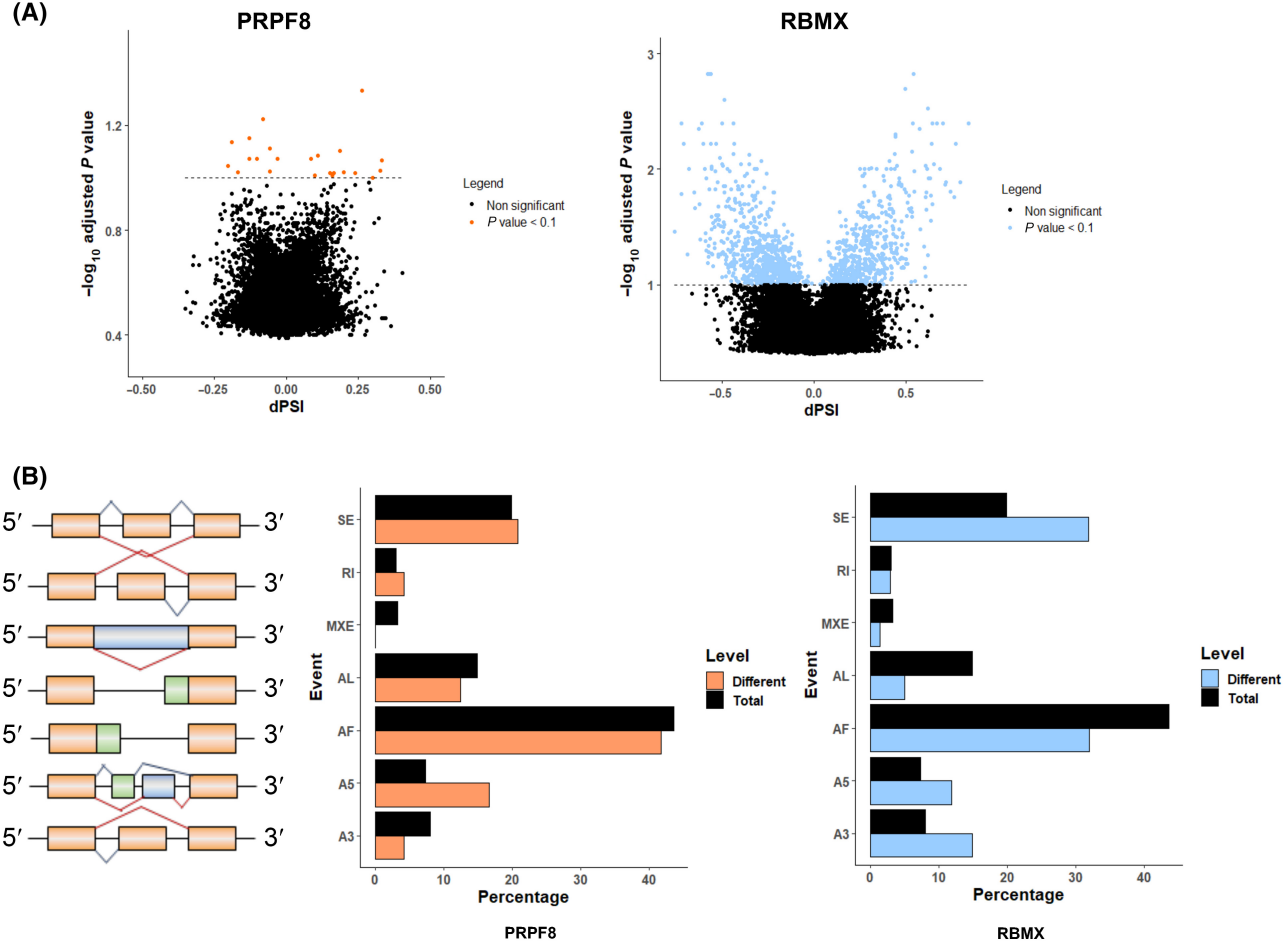
Relationship of *PRPF8* and *RBMX* mRNA expression levels with splicing event patterns in PDAC. (A) Volcano‐plot where ΔΨ of total events calculated is plotted against the –log_10_ *P*‐value of the Fisher's Exact Test to assay differential splicing events between high and low *PRPF8* (orange) and *RBMX* (blue) expression groups of samples, showing their alternative splicing pattern. (B) Alternative Splicing events characterization of RNA‐seq samples. Total splicing events detected (black) and significantly different events between PRPF8 (orange) and RBMX (blue) expression groups are classified depending on their type, showing different frequencies (%) between both conditions. A5/A3, alternative 5′/3′ splice sites; AF/AL, alternative first/last exons; MXE, mutually exclusive exons; RI, retained intron; SE, skipping exon.

We next sought to examine the possible influence of *PRPF8* and *RBMX* on the splicing process in PDAC. To this end, the 94 samples from the “Verona cohort” (cohort 4, see Table 1) were classified in two groups according to their low or high *PRPF8* and *RBMX* mRNA expression level, and the software suppa2 [27] was employed to analyze the number and nature of splicing events in the RNA‐seq. This revealed than only a reduced set of 24 events occurred differentially between low‐ and high‐expressing *PRPF8* samples, while a much larger number, 1324 events, differed in relation to *RBMX* (Fig. 4A). Moreover, whereas the profile of splicing events did not reveal major differences depending on *PRPF8* expression, except for a higher 5′ alternative splice site, samples with high or low levels of *RBMX* expression displayed strikingly distinct patterns of splicing events, with higher frequency of exon skipping, and 5′ and 3′ alternative splice site, and lower frequency of alternative first and last exon, as compared to the average of all the calculated events (Fig. 4B).

### 3.3 Splicing alterations are associated with key PDAC gene mutations

**Fig. 5:**
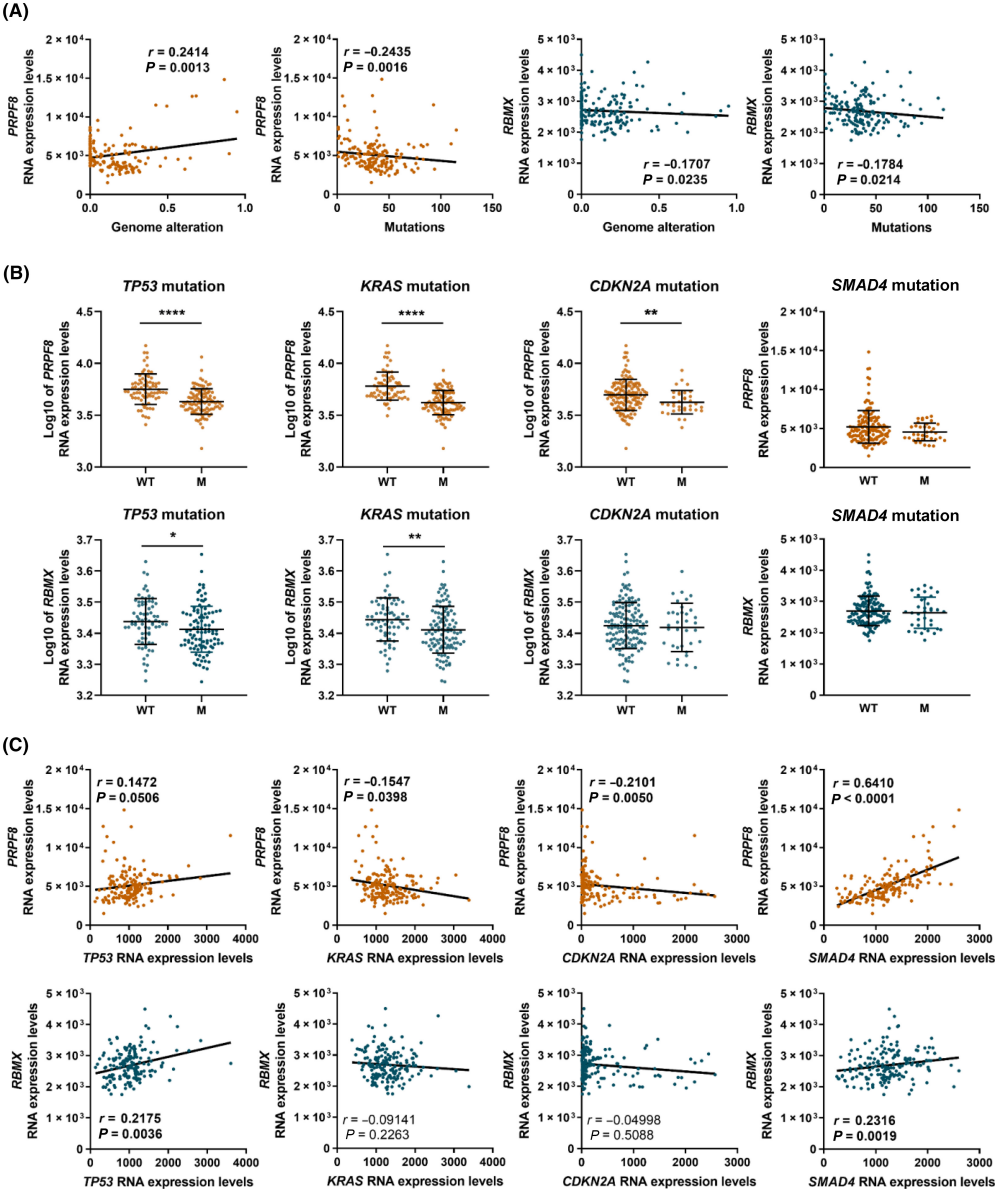
Relationship of *PRPF8* and *RBMX* mRNA expression levels with expression and mutations of key genes in PDAC. (A) Spearman correlations between *PRPF8* (orange) and *RBMX* (blue) mRNA expression levels and Genome alteration and Mutations in PanCancer cohort. (B) Correlations between *PRPF8* (orange) and *RBMX* (blue) Log_10_ mRNA expression levels and *TP53*, *KRAS*, *CDKN2A* and *SMAD4* mutations in PanCancer cohort. Median and interquartile range are represented. Asterisks indicate significant differences between groups by Mann–Whitney *U* test (*P < 0.05; **P < 0.01; ****P < 0.0001). (C) Spearman correlations between *PRPF8* (orange) and *RBMX* (blue) mRNA expression levels and *TP53*, *KRAS*, *CDKN2A*, and *SMAD4*, mRNA expression levels in PanCancer cohort.

Given the preeminent role in PDAC development and progression of mutations in key genes, namely *KRAS*, *TP53*, *CDKN2A*, and *SMAD4*, some of which have already been pathologically linked to altered splicing mechanisms [3], we next evaluated the potential association between *PRPF8* and *RBMX* RNA expression levels and mutations and expression levels of those genes in the PanCancer dataset used earlier. Interestingly, *PRPF8* and *RBMX* expression levels moderately correlated with the overall level of genome alteration and with the above‐mentioned gene mutations (Fig. 5A,B). More specifically, tumors from patients harboring *TP53* and *KRAS* mutations displayed lower *PRPF8* and *RBMX* levels, while *CDKN2A* mutation was related with lower expression of *PRPF8*. No correlation between *RBMX* expression and *CDKN2A* mutation was observed. (Fig. 5B). Further analysis indicated that *PRPF8* and *RBMX* expression levels slightly correlated directly with *TP53* and *SMAD4* levels and, in the case of *PRPF8*, inversely with *KRAS*, and *CDKN2A* (Fig. 5C).

### 3.4 *PRPF8* and *RBMX* are directly correlated with *in vitro* features

**Fig. 6:**
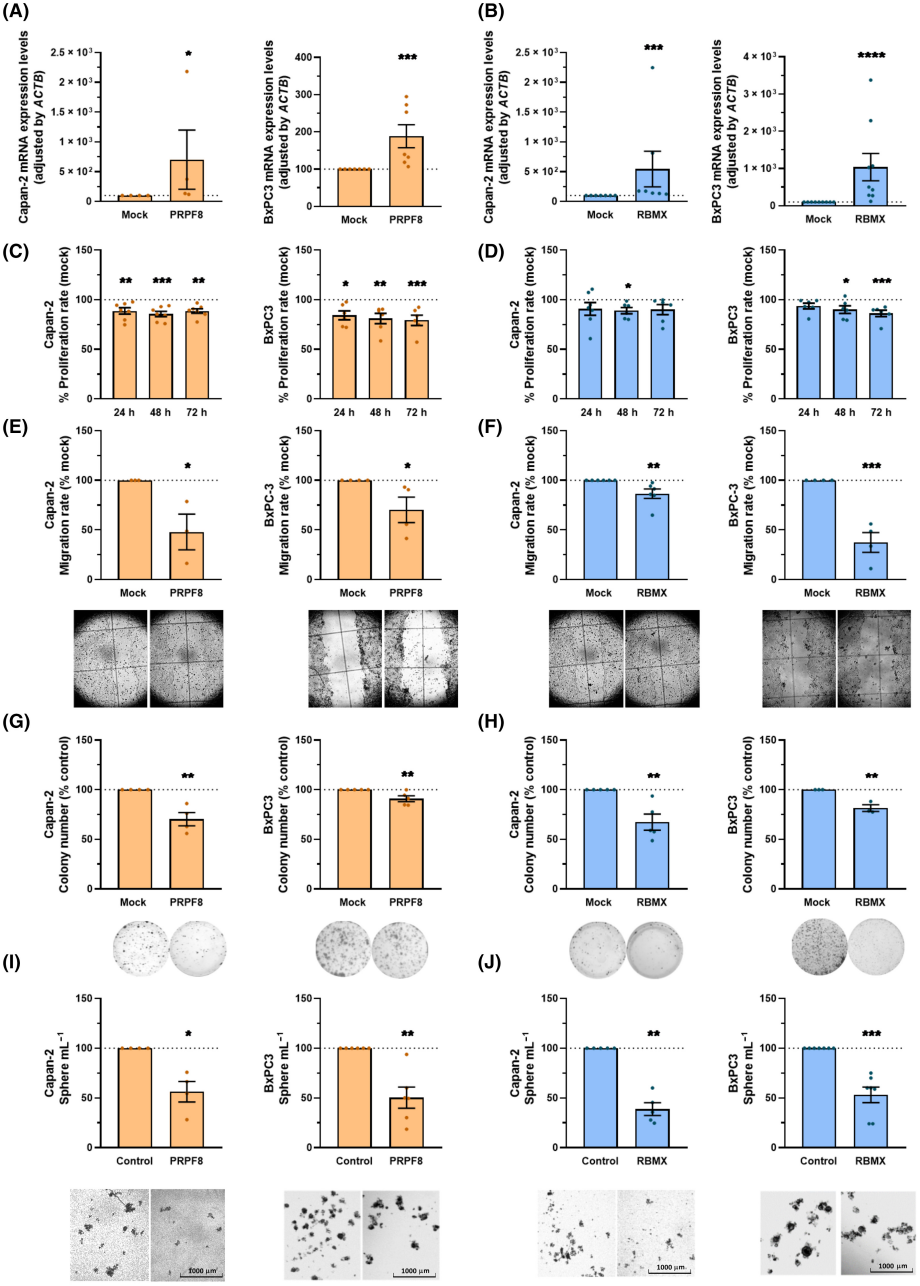
Effect of PRPF8 and RBMX modulation in PDAC. (A, B) RNA expression levels of *PRPF8* (A) and *RBMX* (B) measured in Capan‐2 (*n* = 4; *n* = 7; respectively) and BxPC‐3 (*n* = 7; *n* = 9) cell lines after overexpression with their respective plasmid compared with mock (control; set at 100%). (C, D) Proliferation rates of Capan‐2, and BxPC‐3 cell lines after *PRPF8* (*n* = 7; *n* = 6) and *RBMX* (*n* = 6; *n* = 6) overexpression respectively at 24, 48 and 72 h compared with mock (control; set at 100%), represented as a dot line. (E, F) Migration rates of Capan‐2, and BxPC‐3 cell lines after *PRPF8* (*n* = 3; *n* = 4) and *RBMX* (*n* = 6; *n* = 4) overexpression respectively compared with mock (control; set at 100%), for 24 h. Representative images of wound closures. (G, H) Colony formation capacity of Capan‐2, and BxPC‐3 cell lines after *PRPF8* (*n* = 4; *n* = 5) and *RBMX* (*n* = 5; *n* = 3) overexpression respectively compared with mock (control; set at 100%). Representative images of colony formation. (I, J) Sphere formation capacity of Capan‐2, and BxPC‐3cell lines after overexpression of *PRPF8* (*n* = 4; *n* = 6) and *RBMX* (*n* = 5; *n* = 7) respectively compared with mock (control; set at 100%). Representative images of spheres. Data represents mean ± SEM. Asterisks indicate significant differences between groups by Mann–Whitney *U* test (*P < 0.05; **P < 0.01; ***P < 0.001; ****P < 0.0001).

Given the altered expression of *PRPF8* and *RBMX* in PDAC, and their association with altered splicing and pathological‐molecular characteristics, we set to explore the effects of *PRPF8* and *RBMX* expression modulation in PDAC cell lines. Since a lower expression of both splicing components was found in tumor tissue compared to non‐tumor tissue, we first confirmed low expression levels of both splicing factors in two widely used PDAC model cell lines, Capan‐2 and BxPC‐3 cell lines (Fig. 6A,B). Also, comparison with the non‐tumoral pancreas‐derived HPDE E6E7 cell line showed lower levels of both factors in Capan‐2 and BxPC‐3 cell lines (Fig. S3A). Then, we overexpressed *PRPF8* or *RBMX*, using specific expression plasmids, to rescue their presence in non‐tumoral pancreas. Validation of *PRPF8* overexpression confirmed a substantial increase in Capan‐2 (over six‐fold), and a more modest but appreciable rise in BxPC‐3 (over 70%) in comparison with empty plasmid (mock) transfected cells (Fig. 6A). Similarly, *RBMX* overexpression was confirmed with substantial increases in both Capan‐2 (over 10‐fold), and BxPC‐3 (over 100%) compared to their respective control (mock transfection; Fig. 6B). Western blot analyses confirmed that overexpression with plasmid increased protein levels of PRPF8 and RBMX, although it only reaches statistical differences in the case of BxPC3 transfected with PRPF8 plasmid, and Capan‐2 transfected with RBMX, whereas in the other cases, numerical increases were observed (Fig. S3B,C). Nevertheless, it is worth noting that there is a drastic difference between the levels of both PRPF8 and RBMX in HPDE E6E7 cells in terms of gene expression measured by qPCR and the protein levels observed with western blot, with the latter not differing from those found in BxPC3 and Capan‐2. Interestingly, even though the expression levels of both factors remain substantially elevated over time after transfection, the RNA levels of both factors progressively declined (Fig. S4). This decrease was more pronounced for *PRPF8*, whose expression dropped at 48 h after transfection.

In line with our predictions, the overexpression of *PRPF8* or *RBMX* decreased cell proliferation after transfection. Specifically, a clear, rapid (24 h) and sustained (up to 72 h) decrease was observed in both cell lines after overexpression of *PRPF8*, whereas the effect of *RBMX* upregulation was observable only at 48 h in both cell lines and was long‐lasting (72 h) only in BxPC‐3 cells (Fig. 6C,D). Interestingly, *PRPF8* and *RBMX* overexpression also impacted on cell migration, which was clearly reduced after 24 h as assessed by a wound‐healing assay (Fig. 6E,F). Moreover, *PRPF8* and *RBMX* overexpression impaired colony formation (10–40% reduction) in Capan‐2 and BxPC‐3 cell lines compared to their respective controls (Fig. 6G,H), and reduced severely the formation of tumorspheres in both cell lines (Fig. 6I,J).

## 4 Discussion

There is increasing evidence that PDAC, like many other cancers, features severe alternative splicing dysregulation, causing changes that can contribute to its development and progression [3, 4, 5, 10, 12, 28]. Such dysregulation may often derive from alterations in the machinery that controls the splicing process, comprised by the spliceosome core and ancillary splicing factors, which lead to aberrant expression of RNAs and/or proteins that, in turn, impart oncological features to malignant cells. Specifically, both biocomputational and experimental studies strongly support that spliceosome‐related defects due to altered expression and/or mutation in splicing machinery components may play an important role in PDAC progression [3, 10, 14, 19]. In the present study, the analysis of a comprehensive landscape of splicing machinery elements revealed its broad dysregulation and led to exploring the specific role of two splicing factors, whose downmodulation may play a role in PDAC aggressiveness.

In PDAC—unlike other cancers—assessing the molecular differences between tumor tissue and non‐tumor adjacent tissue can provide meaningful, precise information of changes taking place in pancreatic cells during cancer development [2, 29, 30]. In this regard, our results confirm and extend previous data, by demonstrating a profound alteration in the expression profile of numerous splicing machinery components in PDAC. This phenomenon involved more than one‐third of the spliceosome components and splicing factors examined, whose expression differed in tumor samples vs. adjacent tissue. The changes involved factors from different molecular families with distinct functions on the splicing process (e.g., RNUs, SRSFs, PRPFs, RBMs, etc.), suggesting that the alterations in the splicing machinery in PDAC are not restricted to a limited set of factors, but may rather influence alternative splicing as a whole. These findings lend further support to the rising notion that the splicing machinery is profoundly altered in many diseases, particularly in cancer, although the overarching mechanisms driving this alteration and its overall significance remain to be elucidated. Integrative biocomputational studies on available databases can provide valuable information to this end [31, 32, 33], but specific experimental studies are mandatory to assess the contribution of specific dysregulated molecular components.

Our simultaneous assessment of multiple splicing machinery‐related factors allowed the identification of concurrent changes in expression levels of several altered molecules, which may hold diagnostic and/or prognostic significance. In this regard, on the basis of the substantial value and robustness of the results generated, the use of the adjacent non‐tumor tissue as a control can be considered as the most appropriate, albeit not ideal, due to the increase in glycolytic activity, lactate transport and tumor growth observed in this tissue [34, 35, 36]. This idea is suggested by the combined ability of these factors to discriminate between tumoral and non‐tumor tissue (as indicated by the significant ROC curves) and by their association with critical clinical features, including patient survival. Thus, our findings unveil a set of factors with the potential to enhance the arsenal of molecular biomarkers and targets to tackle PDAC. To bring this concept forwards, we selected two factors, *PRPF8* and *RBMX*.

*PRPF8* (Pre‐MRNA Processing Factor 8, also known as Prp8) is the largest and evolutionarily most conserved protein component of the spliceosome, where it is a component of the snRNP U5 complex [37, 38]. Here, its expression in PDAC tissue was lower than in the adjacent non‐tumor tissue, suggesting both, a potential value as a biomarker and a possible pathological role in this cancer. Mutations in *PRPF8* have been implicated in the development of Retinitis Pigmentosa [39], but the role of this factor in cancer is less well understood, with only some studies reporting its ability to reduce cell growth in colorectal cancer [40] and to modify androgen receptor levels in prostate cancer [41]. Notably, our data show that *PRPF8* levels progressively decreased in higher grade tumor, and that reduced *PRPF8* expression levels in PDAC were remarkably associated with poorer outcome in disease‐specific and overall survival.

*RBMX* (RNA‐binding motif protein, X‐linked, also known as *HNRPNG*) is an essential splicing factor that participates in exon addition or exclusion in the mRNA for many proteins [42]. This factor plays multiple roles in key biological processes, from nervous system development to transcription control, chromosome biology [43], cell division [44] and DNA stability [45]. In cancer, RBMX also exerts relevant actions, which seem to vary diametrically depending on the type of tumor, behaving as either a tumor promoter or a tumor suppressor. Thus, while its overexpression has been related with hepatocellular carcinoma [46] or T‐Cell Lymphomas [47], downregulation is observed in bladder [48], endometrium [49] or neck cancer [50]. In line with the latter, we found that *RBMX* expression is lower in PDAC tumor tissue compared with non‐tumor tissue, a reduction that is associated to lower survival rates (disease specific and overall survival probability) of the patients. However, the association of *RBMX* expression with tumor histological grade is counterintuitive, as it does not have a progressive decrease (like that from *PRPF8*), but an apparent increase in grade 2 tumors compared to grade 1. Our data do not offer a reasonable explanation for this, and we could only speculate that *RBMX* loss may occur early during PDAC development, its expression subsequently fluctuating along histological progression. Clearly, further studies will be required to elucidate this observation.

These observations suggest a splicing‐related role for *PRPF8* and *RBMX* in PDAC, as a reduction in the expression of core spliceosomal components and splicing factors may alter spliceosomal activity. Moreover, the two factors appear to be closely related, as evidenced by the close interaction cluster of STRING and the consistency in the impact on patient survival when both factors are considered. To provide the necessary experimental demonstration, we explored the functional consequences of manipulating the expression of *PRPF8* and *RBMX* in PDAC cell lines. As expected, overexpression of *PRPF8* and *RBMX* in two different PDAC cell lines, restoring their respective levels to mimic those in non‐tumor reference tissues, showed similar results. Specifically, overexpression of *PRPF8* and *RBMX* affected cancer cell lines behavior by reducing proliferation and migration, similar to what was previously reported in comparable experimental settings [6, 7, 9, 10]. Remarkably, restoring the expression of both splicing factors also inhibited sphere and colony formation, indicating that their role might extend to the control of self‐renewal and stem properties [51]. These findings underscore the powerful functional consequences of alterations in splicing machinery components and suggest that its exogenous manipulation could provide means for therapeutic intervention, as we and others have recently proposed in PDAC and other cancers [4, 5, 6, 7, 8, 9, 10, 12, 52]. Specific examples include the use of an anti‐splicing drug in PDAC models by our group [10], the recent links of dysregulated splicing factors with PDAC metastasis [19] and pancreatitis [53], or the use of antisense oligonucleotides to switch alternative splicing patterns in PDAC preclinical models [54].

The relevant role of *PRPF8* and *RBMX* as components of the splicing machinery prompted us to examine the possible implications of their altered expression in RNA splicing in PDAC, by comparing global splicing patterns in tumors with low and high *PRPF8* and *RBMX* expression. Interestingly, this approach revealed clear differences that, in the case of *PRPF8*, and given its core role, were of an unexpected, limited extent. These differences are reflected in the distinct splicing patterns observed, which mainly affected exon skipping and alternative first and last exon. These results suggest that, despite its central implication in common gene processing, PRPF8 may exert its tumor suppressor actions in PDAC by modulating a limited number of splicing events, which certainly deserve a close inspection in the future [55]. Conversely, altered RBMX correlated with changes in the splicing of multiple genes of different families and would therefore implicate different players and mechanisms. In this context, it is worth noting that PDAC samples with lower levels of *PRPF8* and *RBMX* display mutational signatures linked to poorer PDAC prognosis, including mutations of key driver genes as *KRAS* and *TP53* [2, 56, 57]. Likewise, transcriptional analyses linked *PRPF8* and *RBMX* expression with that of key PDAC genes, by showing a direct correlation between both genes with *TP53* and *SMAD4* two key tumoral suppressor genes in PDAC, and an inverse correlation of *PRPF8* expression with *KRAS* and *CDKN2A* mRNA levels [2, 3]. These observations are in line with and provide further support to the recent notion that dysregulations of components of the splicing machinery may exert their actions in connection to altered functioning of well‐recognized key gene players in PDAC like KRAS and P53 [3].

## 5 Conclusions

Taken together, our results demonstrate that the splicing machinery is severely dysregulated in PDAC, where we identified two specific components, *PRPF8* and *RBMX*, that display a downregulated expression closely linked to poorer survival and clinical and molecular markers of bad prognosis, such as *KRAS* or *TP53* mutations. Furthermore, we found that expression of *PRPF8* and *RBMX* is distinctly associated to altered splicing profiles in PDAC, and restoring their expression levels rescued their tumor suppressor ability in two *in vitro* PDAC cell models, by reducing cell proliferation, migration, and colony and tumorspheres formation. We conclude that the splicing machinery is profoundly altered in PDAC, which provides a novel pathway to identify new potential biomarkers and actionable therapeutic targets for this dismal cancer.

## Conflict of interest

The authors declare no conflict of interest.

## Author contributions

EAP: Conception, Data curation, Formal analysis, Investigation, Methodology, Validation, Visualization, Writing – original draft, Writing – review & editing. RBE: Investigation, Methodology, Data curation, Formal analysis, Investigation, Writing – review and editing. MTMM: Investigation, Methodology, Data curation, Formal analysis, Investigation, Writing – review and editing. VGV: Investigation, Methodology, Data curation, Formal analysis, Investigation, Writing – review and editing. JMJV: Methodology, Formal analysis, Investigation, Writing – review and editing. AM: Methodology, Formal analysis, Validation, Writing – review & editing. IGB: Methodology, Formal analysis, Validation, Writing – review & editing. CL: Methodology, Formal analysis, Validation, Writing – review & editing. JMSH: Methodology, Formal analysis, Validation, Resources, Writing – review & editing. MESF: Methodology, Formal analysis, Validation, Resources, Writing – review & editing. ARR: Methodology, Formal analysis, Validation, Writing – review & editing. SPA: Formal analysis, Methodology, Writing – original draft, Writing – review & editing. AV: Methodology, Formal analysis, Validation, Writing – review & editing. MDG: Formal analysis, Methodology, Resources, Writing – original draft, Writing – original draft, Writing – review & editing. RTL: Methodology, Formal analysis, Validation, Writing – review & editing. AS: Data curation, Formal analysis, Investigation, Methodology, Validation, Resources, Writing – review & editing. AAS: Conception, Data curation, Formal analysis, Investigation, Methodology, Validation, Resources, Writing – review & editing. RML: Conception, Data curation, Formal analysis, Funding acquisition, Investigation, Methodology, Project administration, Resources, Supervision, Visualization, Writing – original draft, Writing – review & editing. AIC: Conception, Data curation, Formal analysis, Funding acquisition, Investigation, Methodology, Project administration, Resources, Supervision, Visualization, Writing – original draft, Writing – review & editing. JPC: Conception, Data curation, Formal analysis, Funding acquisition, Investigation, Methodology, Project administration, Resources, Supervision, Visualization, Writing – original draft, Writing – review & editing.

### Peer review

The peer review history for this article is available at https://www.webofscience.com/api/gateway/wos/peer‐review/10.1002/1878‐0261.13658.

## Supporting information


**Fig. S1.** Top splicing factors mRNA expression profile in PDAC.
**Fig. S2.** Distribution of the RNA expression of the nonselected splicing factors among the different histological grades of PDAC.
**Fig. S3.** Expression of PRPF8 and RBMX in model cell lines.
**Fig. S4.** Expression of *PRPF8* and *RBMX* in model cell lines after plasmid transfection over time.

## Data Availability

The datasets used and/or analyzed during the current study are available from the corresponding authors (justo@uco.es, b12ibcoa@uco.es) on reasonable request.
