# Supplementary material for: Spliceosomic dysregulation in pancreatic cancer uncovers splicing factors PRPF8 and RBMX as novel candidate actionable targets
Source: Mol Oncol. 2024 May 24;18(10):2524–40. doi: 10.1002/1878-0261.13658 (PMC11459039; doi:10.1002/1878-0261.13658)
Supplement: Supplementary file 1 — Fig. S1. Top splicing factors mRNA expression profile in PDAC. Fig. S2. Distribution of the RNA expression of the nonselected splicing factors among the different histological grades of PDAC. Fig. S3. Expression of PRPF8 and RBMX in model cell lines. Fig. S4. Expression of PRPF8 and RBMX in model cell lines after plasmid transfection over time. [file MOL2-18-2524-s001.zip › MOL2_13658-sup-2_Supplementary information legends.docx]

**Supporting information**

**Fig S1. Top splicing factors mRNA expression profile in PDAC.** A) Unsupervised hierarchical clustering (euclidean) analysis of mRNA expression levels of the 7 spliceosome components that best contribute to the distinguishability between PDAC FFPE samples (orange) compared with non-tumor adjacent tissue (blue). B) mRNA expression levels of selected splicing machinery components in PDAC FFPE samples compared with non-tumoral adjacent tissue. Data are represented by log10 of mRNA levels normalized by *ACTB* expression levels. C) ROC curve analysis of selected splicing machinery components in PDAC FFPE samples compared with non-tumoral adjacent tissue. D) Integrated ROC curve combining the most significant dysregulated splicing machinery components (*PRPF8, SND1, TIA1, ESRP2, HNRNPA2B1, RBMX, RNU1, SRSF4, MBNL2,* and *TRA2B*) Data represents mean ± SEM. Asterisks indicates values that significantly differences between groups (*, *p* < 0.05; **, *p* < 0.01; ***, *p* < 0.001; ****, *p* < 0.0001).

**Fig S2. Distribution of the RNA expression of the non selected splicing factors among the different histological grades of PDAC.**

**Fig S3. Expression of PRPF8 and RBMX in model cell lines.** A) RNA expression levels of *PRPF8* (top) and *RBMX* (bottom) measured in HPDE, BxPC-3 and Capan-2 cell lines (n = 4). *PRPF8* and *RBMX* expression levels were normalized using normalization factor, calculated with *ACTB* and *GAPDH* expression levels. B) Western blot analyses of PRPF8 (top) and RBMX (bottom) protein expression levels in HPDE, BxPC-3 (transfected with both mock and PRPF8/RBMX plasmids) and Capan-2 (transfected with both mock and PRPF8/RBMX plasmids) cell lines (n = 4). Protein expression levels were normalized using TUBB protein expression levels. C) Representative images of Western blot in B of PRPF8 (top) and RBMX (bottom) experiments. Data represents mean ± SEM. Asterisks indicates values that significantly differences between groups (*, *p* < 0.05; ****, *p* < 0.0001).

**Fig S4. Expression of *PRPF8* and *RBMX* in model cell lines after plasmid transfection over time.** RNA expression levels of *PRPF8* (A) and *RBMX* (B) measured in Capan-2 (n = 3; left) and BxPC-3 (n = 3; right) cell lines after overexpression with their respective plasmid compared with mock (control; set at 100 %). Expression was measured at 24, 48 and 72 hours after transfection. Data represents mean ± SEM. Asterisks indicates values that significantly differences between groups (*, *p* < 0.05; **, *p* < 0.01; ***, *p* < 0.001).
