# Supplementary figures and images for: Spliceosomic dysregulation in pancreatic cancer uncovers splicing factors PRPF8 and RBMX as novel candidate actionable targets
Source: Mol Oncol. 2024 May 24;18(10):2524–40. doi: 10.1002/1878-0261.13658 (PMC11459039; doi:10.1002/1878-0261.13658)

A

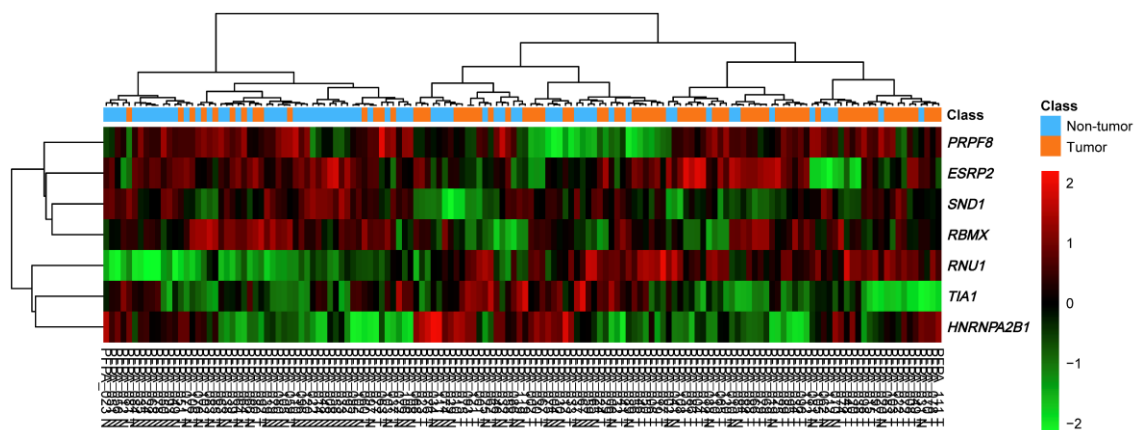

B

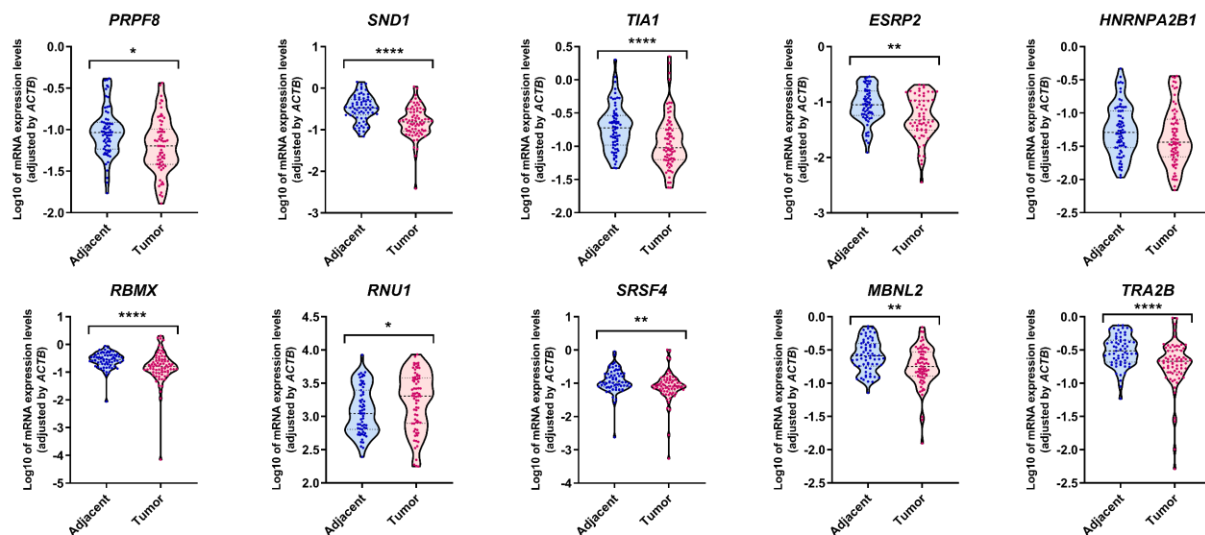

C

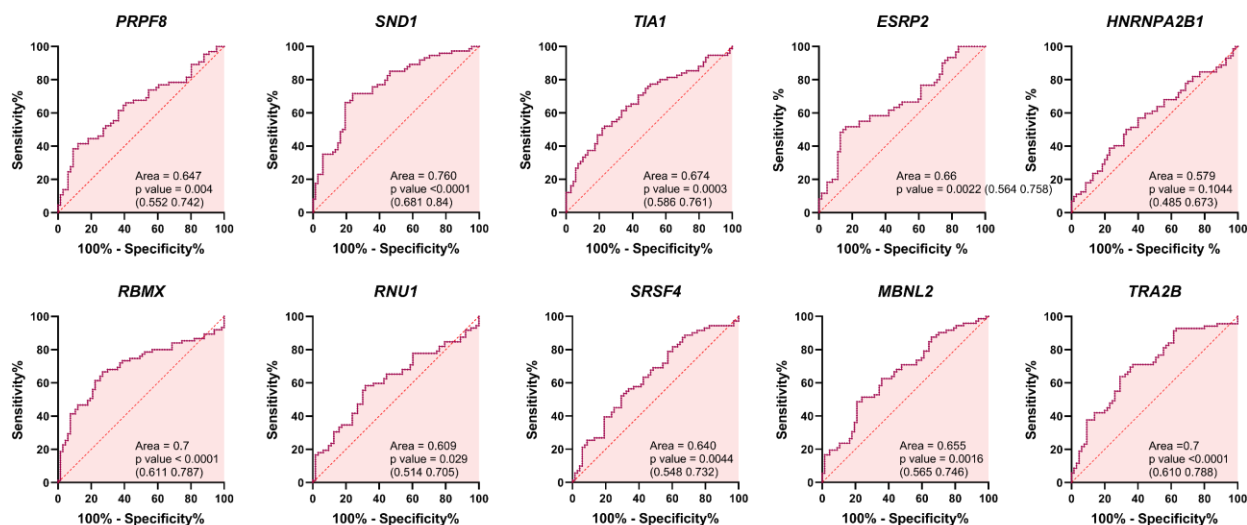

D

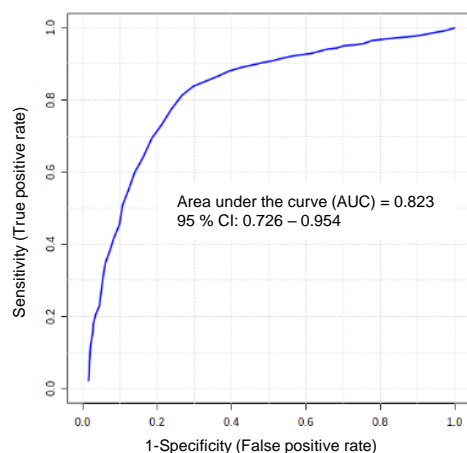

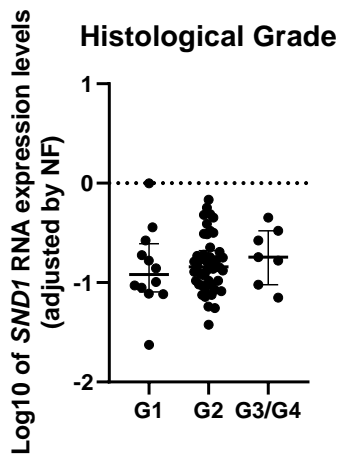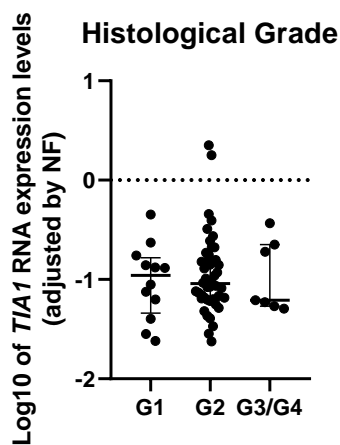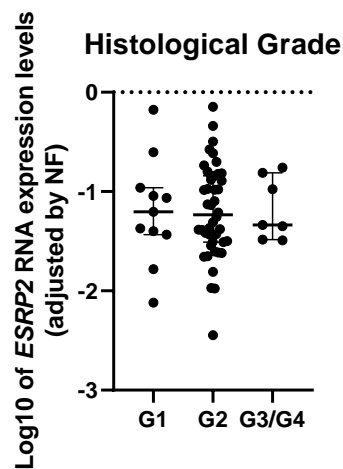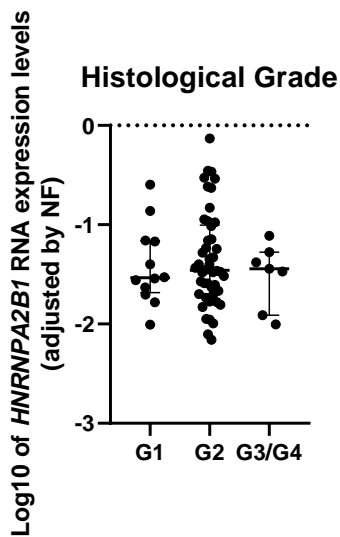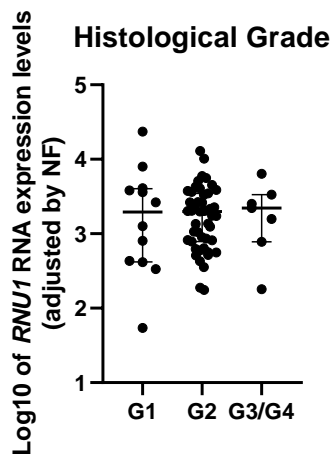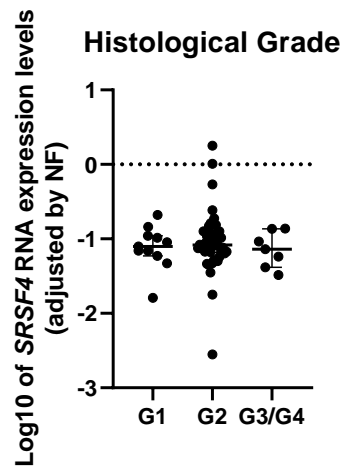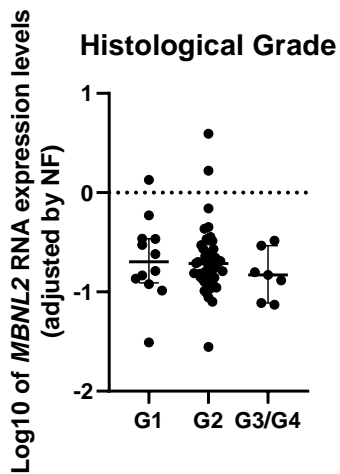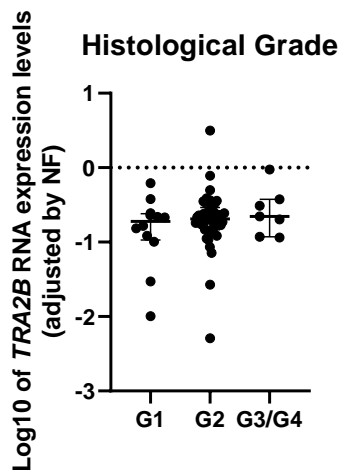

**A)**

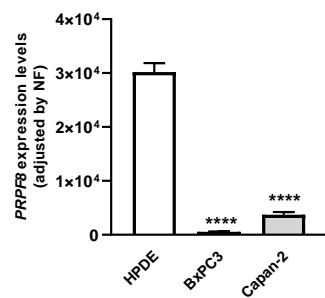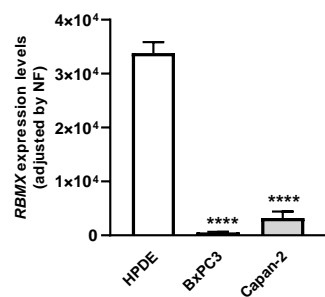

**B)**

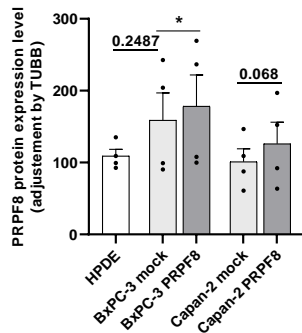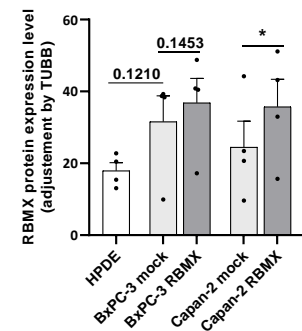

**C)**

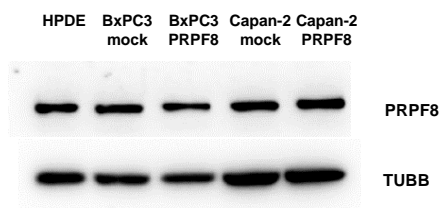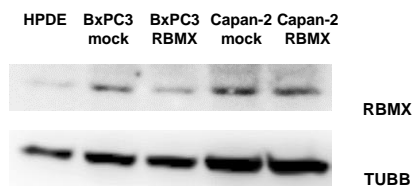

**A**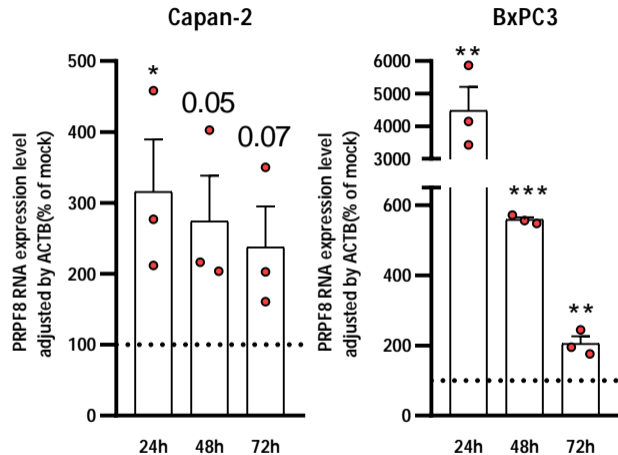**B**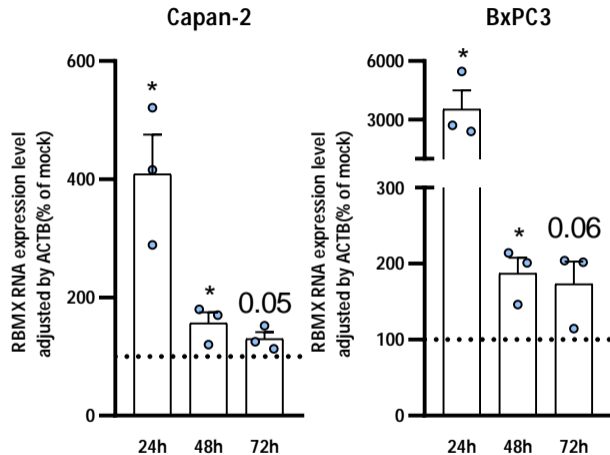

Supplement: Supplementary file 1 — Fig. S1. Top splicing factors mRNA expression profile in PDAC. Fig. S2. Distribution of the RNA expression of the nonselected splicing factors among the different histological grades of PDAC. Fig. S3. Expression of PRPF8 and RBMX in model cell lines. Fig. S4. Expression of PRPF8 and RBMX in model cell lines after plasmid transfection over time. [file MOL2-18-2524-s001.zip › MOL2_13658-sup-1_New Supplemental 2024.pdf]
